# Crystallization of ExoR - His_6_, A Regulatory Signal Controlling Symbiotic Nitrogen Fixation

**DOI:** 10.1063/4.0000837

**Published:** 2025-10-27

**Authors:** Katherine Lee, Kenneth Childers, Madeline Rasche

**Affiliations:** California State University Fullerton, Fullerton, CA, USA

## Abstract

ExoR is a signaling protein that controls nitrogen fixation, the conversion of N2 gas into ammonia fertilizer. The ExoR protein in the soil bacterium *Sinorhizobium meliloti* acts as a novel inhibitor of the two-component regulatory system (ExoS/ChvI), blocking the production of the signaling molecule succinoglycan, which is needed for establishing a nitrogen-fixing symbiosis with the legume. Previous attempts to determine the crystal structure of ExoR have been unsuccessful, due to the tendency of the protein to precipitate at protein concentrations above 3 mg/mL. The goal of the current project is to develop a purification strategy to produce crystallization-quality ExoR protein. Histidine-tagged ExoR (ExoR-His_6_) was produced in *Escherichia coli* and purified in two steps using nickel affinity (NiNTA) chromatography and S200 gel filtration chromatography. After purification, Bradford assay and sodium dodecyl sulfate polyacrylamide gel electrophoresis (SDS- PAGE) showed that the first NiNTA elution sample (100 mM imidazole) contained protein at a concentration of 1.35 mg/mL and a purity of ∼46%. After the S200 gel filtration column, the protein concentration was 0.21 mg/mL, and the purity increased to ∼90%. Altering the collected volumes to modify elution conditions resulted in a 2-fold increase in ExoR concentration. Crystallization using a 1 M ammonium sulfate buffer successfully produced a 100-micron crystal, confirming that a higher protein concentration was benficial for larger, diffraction-quality crystals. Experiments are in progress to increase the purity of the protein to greater than 98%, in order to determine the three-dimensional structure of the protein.

